# MARPE as an adjunct to orthodontic treatment

**DOI:** 10.1590/2177-6709.27.6.e22bbo6

**Published:** 2023-03-27

**Authors:** Orlando TANAKA, Sergio Luiz MOTA-JÚNIOR

**Affiliations:** 1 Pontifícia Universidade Católica do Paraná, Escola de Medicina e Ciências da Vida (Curitiba/PR, Brazil).; 2 Universidade Federal de Juiz de Fora, Curso de Especialização em Ortodontia (Juiz de Fora/MG, Brazil).

**Keywords:** Expansion, Esthetics, Adult treatment, MARPE

## Abstract

**Introduction:**

Miniscrew or microimplant-assisted rapid palatal expansion (MARPE) devices are used to achieve a skeletal expansion of the palate and to increase the arch perimeter.

**Objective:**

To describe the treatment of a 23-year-old woman with an Angle Class II, division 1 malocclusion with constricted maxillary and mandibular arches.

**Case report:**

The patient’s main complaint was mandibular anterior crowding. The treatment plan included expansion of the mandibular arch concurrent with maxillary expansion, using a MARPE appliance in combination with a full-fixed appliance to align and level the crowded mandibular teeth, along with miniscrews as anchorage for the maxillary teeth and for distalization of the molars and premolars. A successful non-extraction orthodontic treatment was accomplished after 28 months, and the occlusion and teeth alignment, as well as facial goals, were resolved in a clinically satisfactory manner.

**Conclusion:**

The treatment objectives were met, and the outcome of the expansion of the maxillary arch with a MARPE appliance as an adjunct to a fixed appliance was considered a success. An esthetic, functional, and stable result after a 1-year follow-up was achieved and was satisfactory to the patient.

## RESUMO

**Introdução:**

Os aparelhos para expansão rápida da maxila (ERM) apoiada em mini-imlantes (MI) (MARPE) são usados para obter uma expansão esquelética do palato e aumentar o perímetro da arcada dentária superior.

**Objetivo:**

Este relato de caso descreve o tratamento de uma paciente do sexo feminino de 23 anos de idade, com má oclusão Classe II de Angle, divisão 1, com constrição das arcadas superior e inferior.

**Relato de caso:**

A queixa principal da paciente era o apinhamento anteroinferior. O plano de tratamento incluiu a expansão maxilar usando o aparelho MARPE, em combinação com aparelho fixo para a expansão da arcada inferior, concomitante ao alinhamento e nivelamento. Utilizou-se MI como ancoragem para a distalização dos molares e pré-molares superiores. O tratamento ortodôntico sem extrações foi realizado em 28 meses, e a oclusão e o alinhamento dos dentes, assim como os resultados faciais obtidos, foram clinicamente satisfatórios.

**Conclusão:**

Os objetivos do tratamento foram alcançados, e o resultado da ERM com aparelho MARPE junto ao aparelho fixo foi considerado um sucesso. Observou-se resultados estéticos e funcionais estáveis após um ano de acompanhamento, satisfatórios para a paciente.

## INTRODUCTION

Rapid palatal expansion (RPE) is used to apply lateral forces to the teeth, which increases the perimeter of the arch and disarticulates the midpalatal suture.^1^ This can be easily achieved in primary or mixed dentition, by expanding the arch with tooth-borne or tooth-tissue-borne appliances,^2^ which relies on a combination of orthopedic and dental expansion to correct the skeletal misalignment.^3^

In adult patients, the midpalatal suture presents with increasingly complex interdigitation, which makes it more challenging to split.^4^ Thus, surgically-assisted rapid maxillary expansion (SARME) is a procedure commonly performed to correct transverse maxillary deficiencies greater than 5 mm, in patients with complete skeletal maturity and closed cranial sutures.^5^

In 2010^6^, RPE reinforced by orthodontic miniscrews (MARPE), positioned on the palatal bone for transverse correction, was introduced; thereby eliminating the need for surgery in patients, and resulted in successful maxillary expansion of the surrounding structures. MARPE emerged as a promising alternative to allow orthopedic expansions without the need for surgical intervention in late adolescence and adulthood.^6^ And, to increase the stability of the miniscrews, it was suggested that the anchorage be bicortical instead of the monocortical anchorage.^7^

This case report presents the treatment of a 23-year-old female patient, with a Class II, division 1 malocclusion with constricted maxillary and mandibular arches. The clinical outcome of the expanded mandibular arch with archwire associated with MARPE, a full-fixed appliance and miniscrews as anchorage for maxillary teeth distalization was successful, as seen at follow-up one year after treatment.

## CASE REPORT

### DIAGNOSIS AND ETIOLOGY

**Figure 1:**
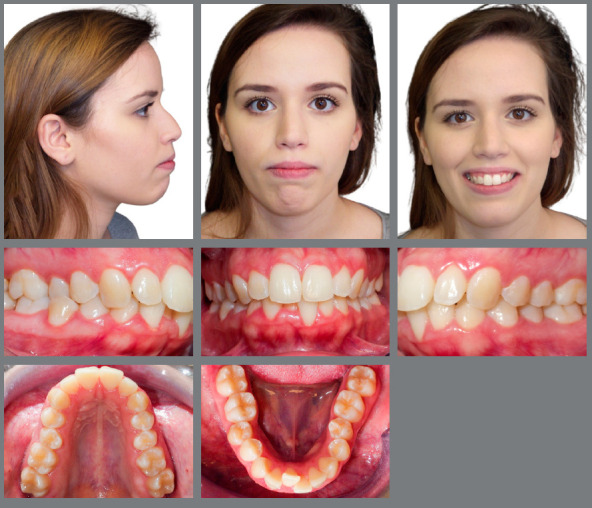
Initial facial and intraoral photographs.

A female patient, aged 23 years and 10 months, sought orthodontic treatment with the main complaint related to esthetic concerns, described as “crowded lower teeth”. She was seeking a second opinion and wished to avoid tooth extraction, as well as any form of surgery. Her general state of health was good, with no contributing medical history. Pre-treatment facial photographs (Fig 1) showed a convex facial profile, with a protruded lower lip. In the front view, a small asymmetry was visible on the right side, which was a bit rounded compared to the left side.

The pre-treatment intraoral photographs (Fig 1) showed a mild gingival recession in the mandibular left central incisor. The posterior teeth presented with a clinically significant palatal inclination and a constricted maxillary arch, with the right second premolar in crossbite. She presented an Angle’s Class II malocclusion, division 1, subdivision, crowding of 2 mm in the mandibular arch, and a 3-mm deep curve of Spee, 6.0 mm of overjet between teeth #11 and #41, and 2.0 mm between teeth #21 and #31, in addition to deep overbite. The width of the maxillary lateral incisors was proportionally smaller than that of the maxillary central incisors. The mandibular midline was deviated 1.5 mm to the right. Gingival recession was visible on the mandibular left central incisor.

**Figure 2:**
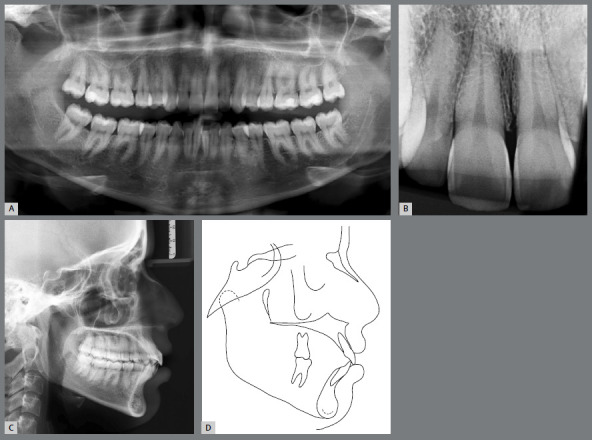
Initial panoramic radiograph **(**A), periapical radiograph **(**B), lateral cephalometric radiograph **(**C) and cephalometric tracing **(**D).

In the panoramic radiograph, all permanent teeth were visible, including extensive restorations in the second molars and tapered incisor root tips (Figs 2A and 2B).

The analysis of the pretreatment lateral cephalometric radiograph and tracings (Figs 2C and 2D), Table 1) revealed a Class II skeletal pattern (ANB = 7º) with a maxillary protrusion (SNA = 87º), protruded mandibular incisors (1.NB = 41º, 1-NB = 11mm and IMPA = 104º), and protruded maxillary incisors (1-NA = 7mm). 

**Table 1:** Pretreatment and post-treatment measurements.

Measurements		Author	Normal	Initial	Final
Skeletal pattern	SNA	Steiner	82°	87°	87°
	SNB	Steiner	80°	81°	81°
	ANB	Steiner	2°	6°	6°
	Witts	Jacobson	♀ 0 ±2mm ♂ 1 ±2mm	3mm	4mm
	Convex. angle	Downs	0°	10°	10°
	Facial	Downs	87,8°	90°	90°
	Y-Axis	Downs	59,9°	60°	60°
	SN-GoGn	Steiner	32°	32°	32°
	FMA	Tweed	25°	25°	25°
	Co-A	McNamara	94-100°	99°	100°
	Co-Gn	McNamara	122-33°	126°	127°
Dental pattern	IMPA	Tweed	90°	104°	102°
	1.NA	Steiner	22°	22°	27°
	1-NA	Steiner	4mm	7mm	4mm
	1.NB	Steiner	25°	41°	35°
	1-NB	Steiner	4mm	11mm	8mm
	Pog-NB	Holdaway		3mm	2mm
	1 - 1	Downs	130°	116°	113°
	1-APog	Ricketts	1mm	6mm	4mm
Profile	LS - S	Steiner	0mm	0mm	0mm
	LI - S	Steiner	0mm	4mm	4mm
	Z-angle	Merrifield	75°	67°	67°

### TREATMENT OBJECTIVES

The patient had a constricted maxillary arch, with mandibular molars and premolars that were lingually inclined as a compensatory mechanism. The first objective, therefore, was to expand the maxillary arch transversely to create an adequate skeletal width, in order to correct the position of the teeth. Additional objectives were to achieve correct overbite and overjet, and to improve the dental and skeletal relationships in the three planes of space.

### TREATMENT ALTERNATIVES

Options for treatment included the following: 1) Maxillary expansion with a Hyrax-type expander, which would require surgery (i.e., surgically-assisted rapid palatal expansion, SARPE); 2) Maxillary expansion with MARPE, in an attempt to avoid surgery; 3) Maxillary expansion with a Hyrax-type palatal expander fixed to the molars and premolars (a non-surgical procedure); 4) Align, level, and carry out dentoalveolar expansion with the orthodontic archwires and intermaxillary elastics; and 5) Perform light interproximal reduction and extraction of four first premolars.

### TREATMENT PROGRESS

**Figure 3:**
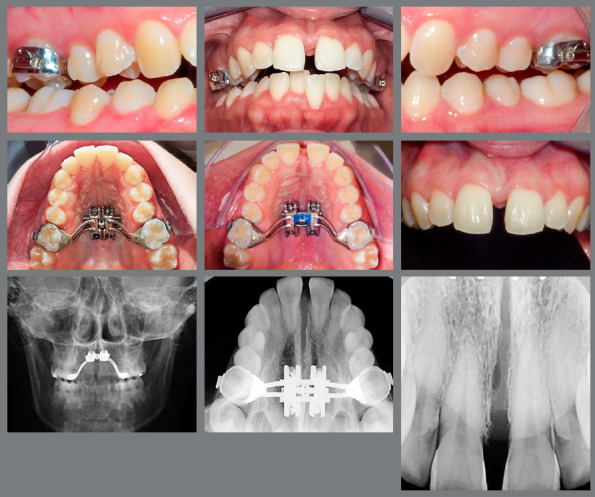
Intraoral and radiograph images after maxillary expansion.

The second option was chosen as the treatment plan for this patient. Treatment was initiated with the placement of a 11.0-mm maxillary skeletal expander (PecLab, Belo Horizonte/MG, Brazil) fixed with four miniscrews (1.8*x*5*x*4 mm anterior and 1.8*x*7*x*4 mm) (Fig 3) and two immediate activations (2/4 of a turn), followed by activations of two turns *per day* for one week. Pain and some discomfort in the palate and nasal cavity areas, as well as headache, was reported by the patient on the fourth day. These issues were resolved by diminishing the expansion to an activation rate of 1 turn *per day* and prescribing an analgesic.

By the tenth day, the patient reported hearing clicks in the region of the palatal suture and, in the following days, reported the appearance of the midline diastema (Fig 3). There was a discrete opening of the anterior bite due to contact of the buccal cuspid of the left first maxillary molar, which moved in the direction of the overlapping mandibular molar. The activations were stopped after 25 turns and the appliance was stabilized. The radiographic image shows the opening of the midpalatal expansion (Fig 3).

Subsequently, brackets were bonded to all teeth, except for the first molars. The following archwires were used: 0.016 x 0.016-in NiTi heat-activated, 0.016 x 0.022-in NiTi, 0.017 x 0.025-in NiTi heat-activated, 0.018 x 0.025-in SS, and 0.019 x 0.025-in SS finishing archwire.

Miniscrews between the second premolars and first molars were applied to distalize the upper left molars and premolars. 

## RESULTS

**Figure 4:**
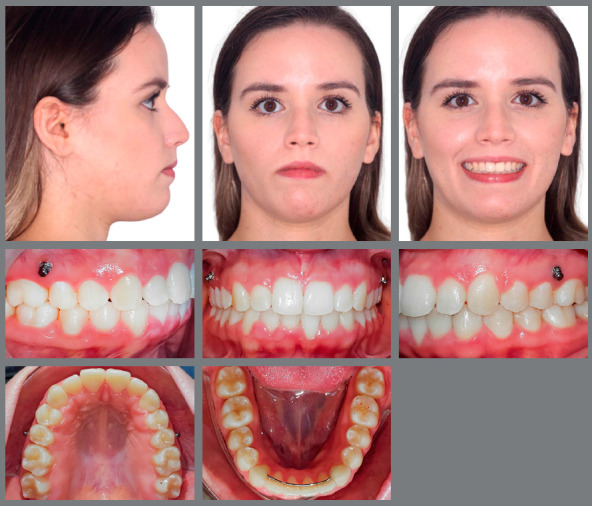
Final facial and intraoral photographs.

**Figure 5:**
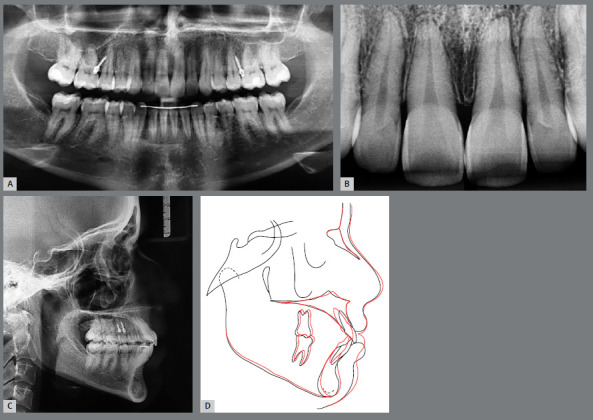
Final panoramic radiograph **(**A), periapical radiograph **(**B), and lateral cephalometric radiograph**(**C). Initial and final tracings superimposition **(**D).

After 28 months of treatment, the esthetic and functional dental and facial goals of the treatment were achieved (Figs 4 and 5). The patient presented with a convex profile and passive lip sealing. The Class II malocclusion was corrected, and the overjet and overbite were satisfactorily reduced. For the retention phase, a wraparound type retainer in the maxillary arch was used and worn full-time for one year, after which it would be required for nighttime use only during subsequent years, to maintain occlusal stability.

**Figure 6:**
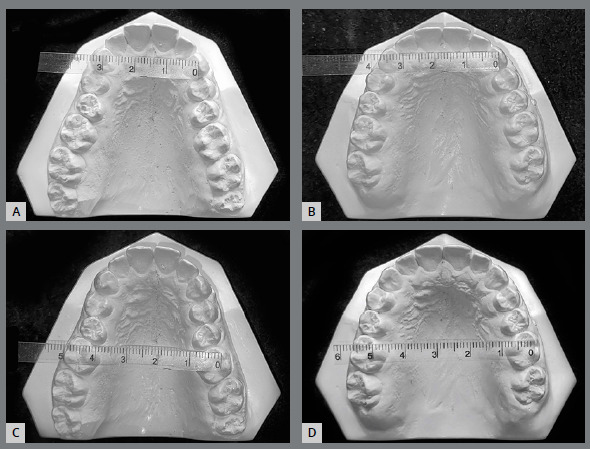
Pretreatment and post-treatment maxillary intercanine **(A,** B) and intermolar **(C,**D) widths.

**Figure 7:**
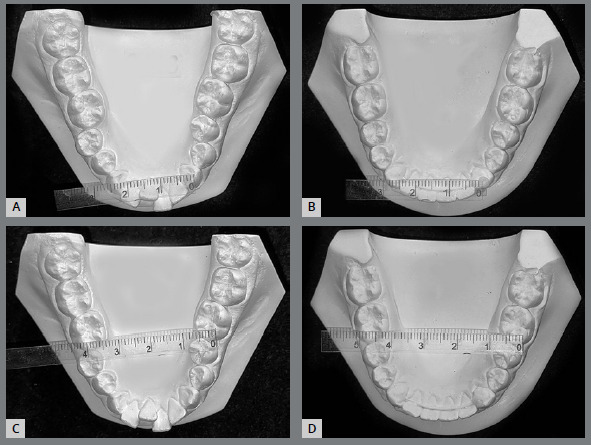
Pretreatment and post-treatment mandibular intercanine **(A,** B) and intermolar **(C,**D) widths.

Satisfactory root parallelism was also observed, as can be seen in Figure 5. Clinically significant expansion in both maxillary and mandibular intermolar and intercanine widths was observed (Figs 6 and 7).

**Figure 8:**
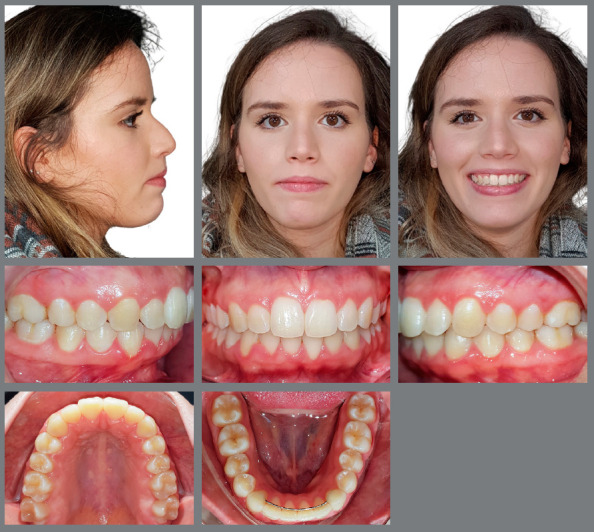
One-year follow-up facial and intraoral photographs.

The Table 1 shows pretreatment and post-treatment cephalometric measurements. Figure 8 shows the results of the 1-year follow-up, where the stability of the occlusal and transverse expansion can be observed.

## DISCUSSION

This case report is not unusual, but represents a clinical situation routinely found in daily practice, and an example of a case in which a posterior crossbite was not an essential clinical condition for undertaking maxillary expansion. The treatment improved the transverse maxillary dental arch dimension with a MARPE appliance during the first stage, followed by mandibular arch expansion during treatment with rectangular archwires, in accordance with McNamara et al.^8^

Rapid maxillary expansion (RME) is typically the standard treatment method for patients presenting transverse deficiency of the maxillary bone. RME can be successfully carried out in young patients who do not have a closed midpalatal suture,^9^ and the treatment can be accomplished with tooth anchorage. 

The adult patient in the present case report did not wish to undergo surgery, that is, she did not want to undergo SARPE. And the RME attempt was not considered due to the uncertainty of the successful outcome. Since MARPE was first described, it has been shown that the maxilla could be expanded with skeletal disjunction and without SARPE.^6^ Therefore, the MARPE approach was chosen and executed, and the post-treatment records demonstrate that a successful result was achieved. The advantages and limitations of non-surgical RPE in adult patients should be individually analyzed to determine the risks and benefits,^10^ as described for the patient reported here.

The selected expander needed to be larger than necessary for the expansion, along with bicortical anchorage (oral and nasal) for achieving the successful outcome described in our case report. The expander selected needs to deliver the maximum expansion capacity and should be kept at an ideal vertical distance from the palatal mucosa, as was achieved in the present case report. If the expander is too distant from the mucosa (more than 2 mm), the miniscrew may fail to reach the nasal cortical bone, as reported by Brunetto et al.^11^

Ricketts et al.^12^ concluded that 1 mm of canine expansion produces 1 mm of arch length increase, and 1 mm of molar expansion results in an increase of 0.25 mm in arch length. Thus, to achieve a good outcome for the non-extraction treatment used in the present case, with a crowded and constricted dental arch, it was necessary to increase the arch perimeter to allow for arch alignment and leveling. 

The present case report showed that maxillary and mandibular arch expansion, followed by a fixed orthodontic appliance, led to increases from 23 mm to 28 mm in intercanine width, and 41 mm to 49 mm in intermolar width for the lower arch. In the upper arch, intercanine distance increased from 32 mm to 35 mm, and intermolar distance from 47 mm to 56 mm. The arch perimeter increased 5.3 mm and 6.0 mm for mandibular and maxillary arches, respectively. Adkins et al.^13^ found an increase of 4.7 mm in the upper arch perimeter after RPE with a Hyrax appliance in an adolescent patient, and McNamara et al.^8^ found an increase from 3 to 4 mm for maxillary arch expansion in children; whereas, Handelman et al.^14^ found those measurements to be 4.5 to 5.5 mm in adults. In the mandibular arch, the example provided by McNamara et al.^8^ increased by only 1 to 2 mm. However, there was a difference in measurement methods: we measured from the tip of the canines, whereas McNamara et al.^8^ used lingual landmarks. In our case, this gain was enough to increase the arch perimeter, correct the enlarged overjet, and solve the problem of mandibular crowding.

We observed a clinically favorable occlusion and esthetic gain in our patient 1 year after treatment. Permanent mandibular retention was chosen due to the strong tendency toward arch-width relapse, as described in the literature.^15^ In addition, mandibular crowding was the patient’s main complaint before treatment.

The present patient showed an improvement in the gingival leveling of the mandibular incisors, resulting from orthodontic alignment and protrusion, and no gingival recession was detected over the long term. Gingival recession associated with orthodontic treatment is a controversial issue, but no association between proclined teeth and gingival recession was found after a 5-year follow-up.^16^ Gingival recession may also be influenced by gingival phenotype, but the present patient had a gingival phenotype that could be classified as optimal.

Overall, simultaneous maxillary and mandibular arch expansion using a nonsurgical approach is a viable procedure for young adults. In selected cases, it can offer a clinically favorable result in the long term. No periodontal disease occurred in this patient, since she presented good oral hygiene.

## CONCLUSION

An adult patient with mildly constricted maxillary and mandibular arches was successfully treated with the MARPE appliance as an adjunct to a fixed appliance. The final outcome was functional occlusion and satisfactory facial esthetics that met the patient’s expectations. The 1-year follow-up showed the stability of the results.
